# Dual Coronary Artery Dissection With Contrasting Healing Patterns: Spontaneous Left Circumflex and Iatrogenic Right Coronary Artery

**DOI:** 10.7759/cureus.74986

**Published:** 2024-12-02

**Authors:** Hassan Elzain, Abdullahi Mohamed Khair, Osama Idris, Nashwa Ahamed, Anas Babiker

**Affiliations:** 1 Cardiology, Royal Care International Hospital, Khartoum, SDN; 2 Cardiology, Amiri Hospital, Kuwait City, KWT

**Keywords:** coronary angiography after coronary dissection, different healing outcomes in coronary artery dissection, healing after scad, mi in young women, pregnancy-related scad

## Abstract

Spontaneous coronary artery dissection (SCAD) is an uncommon but important cause of acute coronary syndrome (ACS), particularly in postpartum women without traditional cardiac risk factors. Our case involves a 29-year-old postpartum woman who presented with severe substernal chest pain eight days after an emergency cesarean section for pregnancy-associated hypertension. Electrocardiography showed ST elevation in the inferior and posterior leads, and coronary angiography revealed a spontaneous dissection in the left circumflex artery (LCx) with an intramural hematoma, alongside a dissection of the right coronary artery (RCA) extending from the ostium to the mid-vessel. This RCA dissection was most likely catheter-induced. Patients with SCAD often have frail arterial walls that are predisposed to dissection, even with minimal trauma, as seen in this case. The patient was treated medically with aspirin, clopidogrel, and bisoprolol. After 10 months, she presented with anginal chest pain and a positive stress ECG. Coronary angiography showed complete healing of the LCx and multiple stenotic lesions in the RCA. This disparity in healing patterns may be attributed to different mechanisms underlying the dissections: spontaneous dissections, which typically involve hormonal and vascular remodeling, versus iatrogenic dissections, which can be influenced by procedural trauma. This case highlights the contrasting healing patterns of spontaneous and iatrogenic dissections and emphasizes the importance of clinical suspicion, procedural caution, and long-term follow-up.

## Introduction

Spontaneous coronary artery dissection (SCAD) is an underappreciated cause of acute coronary syndrome (ACS), particularly in young and postpartum women [1-4]. SCAD is defined by the separation of the coronary artery wall layers, which can result in an intramural hematoma or obstruction of the arterial lumen [4]. This condition is most commonly observed in patients without traditional cardiovascular risk factors, complicating prompt diagnosis [4]. The postpartum period is particularly significant due to a unique combination of hormonal and hemodynamic changes that heighten the risk of SCAD. During pregnancy, elevated levels of estrogen and progesterone play a role in vascular remodeling, leading to a weakening of the arterial wall and reduced collagen content, which contributes to vessel fragility [2-4]. After childbirth, sudden hemodynamic shifts, including increased cardiac output and vascular resistance, impose additional stress on the coronary arteries [2-5]. In the case of our patient, a history of pregnancy-associated hypertension may have exacerbated these conditions, making her more susceptible to dissection [6]. This inherent fragility underscores the importance of careful handling during diagnostic or interventional procedures [7-9].

SCAD is estimated to account for 0.2%-4% of all ACS cases. It is more frequently observed in females, particularly those under 50 years of age, with a reported incidence of 1%-4% of ACS cases in this group [4,10,11]. During pregnancy and the postpartum period, the incidence is even higher, contributing to a significant proportion of ACS cases in this population [5]. Iatrogenic coronary artery dissection is less common, with a reported incidence of less than 0.2% during diagnostic coronary angiographies [7].

In our case, coronary angiography revealed two distinct dissections. The left circumflex artery (LCx) exhibited a spontaneous dissection with an intramural hematoma, while the right coronary artery (RCA) showed a spiral dissection extending from the ostium to the mid-vessel. The RCA dissection was most likely induced by catheter trauma during diagnostic angiography. The different mechanisms of dissection contributed to varying healing outcomes, with the LCx dissection healing completely normally and the RCA dissection evolving into multiple significant stenotic lesions affecting the whole artery.

This case highlights the diagnostic and therapeutic challenges of managing SCAD in the postpartum population, particularly when complicated by iatrogenic factors [4,7]. It underscores the need for heightened clinical suspicion, meticulous procedural techniques, and careful long-term follow-up in such cases [5,7].

## Case presentation

A 29-year-old multiparous woman, eight days postpartum following an emergency cesarean section for pregnancy-induced hypertension, presented to the emergency department with acute substernal severe chest pain accompanied by shortness of breath and sweating. She had no prior medical history of coronary artery disease risk factors, including hypertension, diabetes, smoking, hyperlipidemia, or a family history of premature coronary artery disease or sudden cardiac death. There was no history of connective tissue disorders or use of alcohol, drugs, or oral contraceptives. Her antihypertensive medication had been discontinued after delivery as her blood pressure normalized.

**Figure 1:**
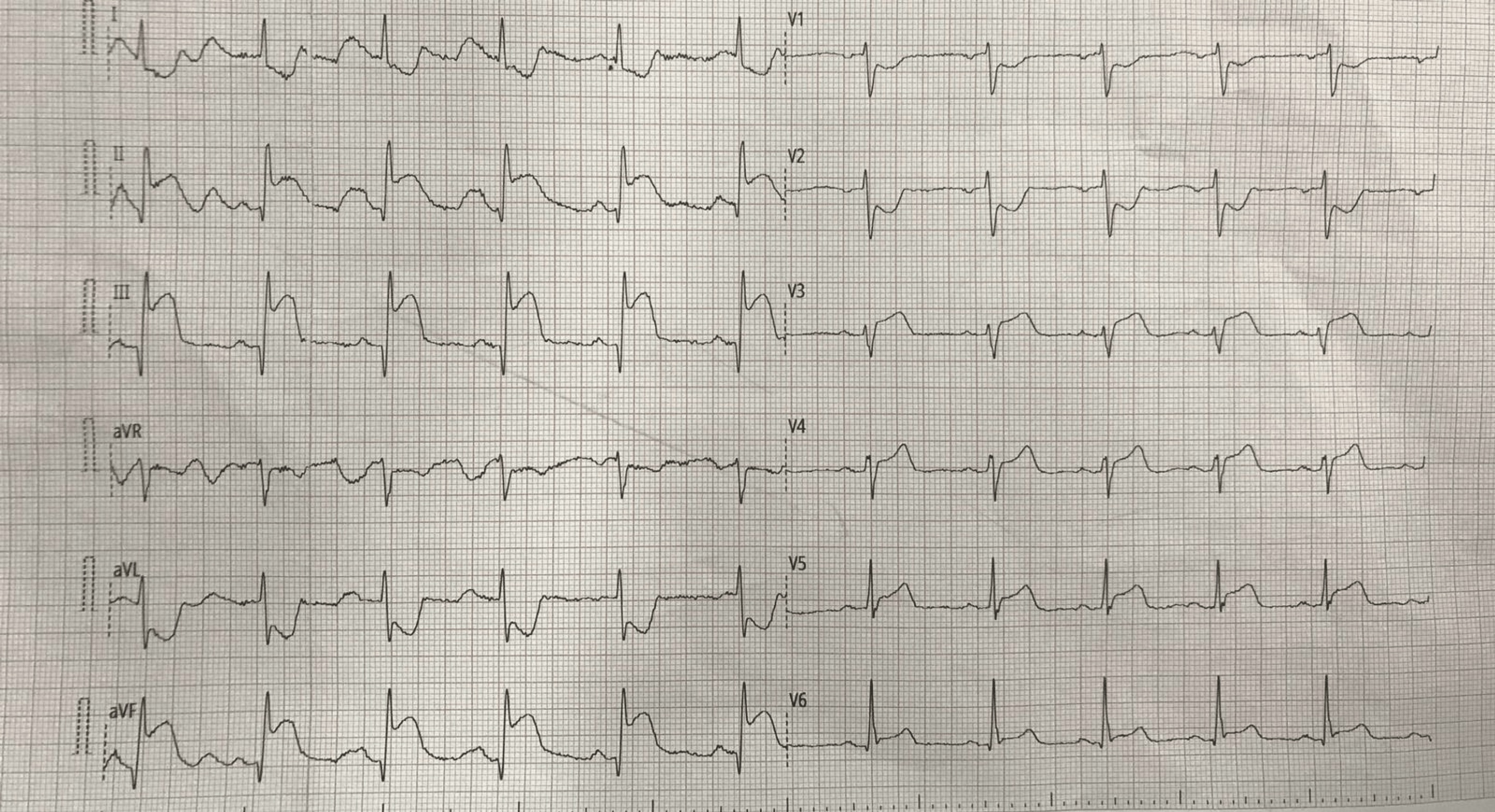
ECG shows inferior ST segment elevation myocardial infarction (STEMI) with posterior extension. Electrocardiography (ECG) demonstrated 2–3 mm ST-segment elevation in leads II, III, and aVF, consistent with inferior wall involvement. Additionally, reciprocal ST-segment depression was observed in leads I and aVL, along with ST depression in leads V1–V2, suggestive of posterior wall involvement.

On physical examination, she appeared acutely distressed, diaphoretic, and in pain. Her heart rate was 110 beats per minute, and her blood pressure was 100/60 mmHg, equal in both arms. A cardiovascular examination revealed a pansystolic murmur at the apex. Chest examination was clear, and there were no signs of deep vein thrombosis. The remainder of the examination was unremarkable (Table 1). Electrocardiography (ECG) showed ST elevation involving the inferior and posterior leads, with reciprocal changes in the high lateral leads (Figure 1). Troponin-T was 6.7 ng/mL (normal reference: less than 0.014 ng/mL). ECG demonstrated mildly impaired left ventricular function with posterior leaflet tethering, resulting in moderate ischemic mitral regurgitation. The posterior leaflet tethering likely reflected ischemic damage to the papillary muscles or left ventricular remodeling secondary to SCAD. These findings are consistent with the coronary angiography results, which revealed LCx dissection. The dissection correlated with the ischemic regions identified and their functional impact on the mitral valve apparatus.

**Table 1:** Laboratory findings. The patient’s laboratory investigations, including a normal lipid profile and HbA1c, were unremarkable, which is consistent with the typical presentation of spontaneous coronary artery dissection in young postpartum women who have no traditional cardiovascular risk factors. LDL: low-density lipoprotein, HDL: high-density lipoprotein, TSH: thyroid-stimulating hormone, HbA1c: glycated hemoglobin.

Test Parameters	Patient Values	Reference Values
Haemoglobin	11.34	12.0-16.0 g/dL
White blood cells	9.49	2.8-10 × 10³/µL
Platelets	213	150-400 x 10³/mL
Blood urea	18	6-24 mg/dL
Creatinine	0.7	0.7- 1.3 mg/dL
Potassium	3.92	3.5 -5.2 mEq/L
Sodium	145	135-145 mEq/L
Triglyceride	123	Below 150 mg/dL
Cholesterol	156	Less than 200 mg/dL
LDL	87	Less than 100 mg/dL
HDL	51	35- 80 mg/dL
TSH	1.27	0.4-4.6 mU/L
HbA1c	4.6	4.5%-5.7%

Coronary angiography revealed a spontaneous dissection of the LCx with an intramural hematoma extending into the distal vessel and the first obtuse marginal branch, with TIMI 3 flow (Video 1).

**Video 1:** Coronary angiography: LCx. Spontaneous LCx dissection with intramural hematoma extending to the distal vessel and OM1 with TIMI3 flow. LCx: left circumflex artery, OM1: first obtuse marginal branch, TIMI3: thrombolysis in myocardial infarction grade 3 flow.

The RCA showed a dissection from the ostium extending to the mid-vessel, most likely catheter-induced, with TIMI 3 flow (Video 2).

**Video 2:** Coronary angiography: RCA. Spiral RCA dissection from the ostium extending to the mid-vessel most likely catheter-induced. RCA: right coronary artery.

The patient was managed conservatively with dual antiplatelet therapy (aspirin and clopidogrel) and a beta-blocker (bisoprolol). Additional supportive measures included pain management and close hemodynamic monitoring. She remained hemodynamically stable throughout her hospital stay and was discharged in stable condition after five days with instructions for follow-up care.

One month later, a follow-up revealed a complete resolution of her symptoms. Echocardiography at that time showed normalization of left ventricular function and resolution of mitral regurgitation. She had resumed normal physical activity without limitations.

Ten months after discharge, the patient returned with anginal chest pain that limited her physical activity and was not adequately controlled with antianginal medications. A stress ECG was performed and was positive, prompting repeat coronary angiography. The angiography revealed that the entire RCA was affected by multiple stenotic lesions (Video 3). The LCx appeared normal, with no evidence of stenosis, and maintained normal flow (Video 4).

**Video 3:** Coronary angiography: 10 months later. Coronary angiography after 10 months showed the RCA healed with multiple stenotic lesions involving the entire artery. RCA: right coronary artery.

**Video 4:** Coronary angiography. Angiographic image of the left circumflex artery (LCx) 10 months after the initial dissection, showing a normal appearance with no evidence of stenosis or abnormal blood flow, indicating complete healing.

## Discussion

SCAD is an uncommon but increasingly identified etiology of ACS, particularly in younger females without traditional cardiovascular risk factors [1,4]. It is frequently linked to precipitating factors such as pregnancy, postpartum hormonal fluctuations, or periods of extreme physical or emotional stress. In this case, the patient developed SCAD eight days following an emergency cesarean section, a timeframe characterized by significant hormonal shifts, vascular remodeling, and heightened physical and emotional stress [3,4]. These factors collectively amplify susceptibility to arterial wall disruption, aligning with the established risk profile of SCAD in postpartum women.

Dual-vessel coronary dissection, as observed in this patient, is relatively uncommon and poses unique diagnostic and management challenges [12]. The simultaneous involvement of the LCx and the RCA underscores the variability in the pathophysiological mechanisms of dissection. The LCx healed spontaneously without any residual stenosis, consistent with the typical healing pattern seen in SCAD cases [1,13]. In contrast, the RCA dissection, which was likely iatrogenic due to the initial coronary angiography, was identified after ten months and demonstrated multiple stenotic lesions, indicating a more complex healing process.

The RCA dissection, which extended from the ostium to the mid-vessel, was most likely catheter-induced during the initial coronary angiography. Iatrogenic dissection is a known complication, especially in patients with fragile coronary arteries like those with SCAD [4,7]. This case highlights the need for meticulous care during coronary interventions to minimize the risk of exacerbating existing vulnerabilities [4,7-9]. While coronary angiography was pivotal for the initial diagnosis, it has limitations in assessing the full extent and characteristics of SCAD [4]. Advanced imaging modalities, such as intravascular ultrasound (IVUS) and optical coherence tomography (OCT), offer detailed visualization of the arterial wall and can delineate dissection flaps and the true and false lumen more accurately [4,8,9,14]. Unfortunately, the unavailability of these techniques at our center limited our diagnostic approach to angiography alone. The use of IVUS or OCT might have provided greater insight, potentially influencing management strategies or enhancing diagnostic confidence in this complex case.

In patients with SCAD, it is essential to take specific precautions during coronary interventions to minimize the risk of iatrogenic dissection. These precautions include using smaller-caliber catheters, handling them gently, and avoiding high-pressure injections. Controlled and careful maneuvers help prevent additional stress or damage to the already fragile arterial wall [4,7,9].

The treatment of SCAD primarily focuses on conservative management, as most cases heal spontaneously over time. Beta-blockers and antiplatelet agents are commonly used as part of medical therapy [13,15,16]. Interventional strategies are generally reserved for cases with persistent ischemia, significant hemodynamic instability, or when symptoms do not improve with medical management [4,5]. Percutaneous coronary intervention (PCI) can be considered but must be approached with great care due to the vulnerability of the affected vessels and the risk of exacerbating the dissection. Coronary artery bypass grafting (CABG) may be necessary for patients with severe coronary involvement or when PCI fails to achieve sufficient relief [16].

The absence of advanced imaging modalities such as IVUS and OCT at our center posed a significant limitation in the assessment of SCAD. These imaging techniques are vital for providing a more comprehensive view of the arterial wall, identifying the true and false lumens, and characterizing dissection flaps with greater accuracy. The inability to utilize IVUS or OCT restricted our diagnostic capabilities to conventional angiography, which, while helpful, may not fully capture the extent of dissection or subtle vessel abnormalities. The integration of these advanced techniques, if available, could have provided a clearer view of the dissection details and the exact condition of the vessel walls.

## Conclusions

This case of SCAD in a postpartum woman underscores several critical points for clinical practice. It contributes to the broader understanding of SCAD by highlighting its presentation in postpartum patients and its management in resource-limited settings where advanced imaging techniques like IVUS or OCT may not be available. The absence of these modalities in this case limited the ability to fully evaluate the dissection, potentially impacting clinical decision-making and patient outcomes.

The long-term follow-up of SCAD patients is essential, especially for those with residual lesions, to monitor for potential complications such as restenosis or recurrent ischemia. This emphasizes the importance of continuous surveillance to ensure patient safety and detect any adverse developments. Clinicians should remain vigilant for the potential of SCAD, particularly in cases involving iatrogenic dissection, due to the possibility of varied healing outcomes. Extreme caution during diagnostic and interventional procedures is crucial to protect the fragile coronary arteries.

This case also highlights the importance of tailored management strategies for complex SCAD presentations. Future research should focus on understanding the long-term outcomes of SCAD and iatrogenic dissection. Studies aimed at exploring these outcomes are essential for informing clinical practice and improving patient care. Enhanced awareness and ongoing education about SCAD are vital for improving patient outcomes and ensuring that clinicians are prepared to manage these challenging cases effectively.

In conclusion, the potential for iatrogenic dissection in SCAD and its unique outcome features necessitate careful, individualized approaches to management. The insights gained from this case can inform both clinical practice and future research, reinforcing the need for meticulous handling, long-term follow-up, and continued investigation into the outcomes of SCAD and iatrogenic dissection.
